# United States Veterinary Medical Association

**Published:** 1890-06

**Authors:** 


### EDITORIAL.

### U. S. VETERINARY MEDICAL ASSOCIATION.

THE Committee of Arrangements for the Chicago meeting September 16th and 17th, 1890, have informed us that they have arranged the following itinerary :

A train will leave New York by the Baltimore and Ohio Railroad on Sunday morning, September 14th, and pass through Philadelphia, after which it runs along the banks of the Delaware River through Chester and Wilmington to Newark, Del., and soon crosses the Susquehanna on the finest steel bridge in the country, one mile and a third long and ninety-seven feet above high water, affording a combined panorama of valley and coast scenery. After crossing to Baltimore on the transfer steamer, with a view of the harbor of this great industrial city, and a sight of Fort McHenry, the trip continues to Washington, 136 miles in three hours. With the addition of the Baltimore and Washington members, the train will start Westward. Crossing the Monocacy, the old Potomac, and passing by Harper's Ferry, the heights of Maryland, Loudon and Bolivar, and the Shenandoah Valley, which are all historical places of the War of the Rebellion, are passed. Within view of South Mountain and through Cumberland, over the country by which General Lee marched to Gettysburg, the trip ascends the Alleghanies to Deer Park, 3000 feet above sea level, and then goes down the Western slope over a most beautiful country to Grafton. At Parksburg, where the Ohio River is crossed, and on through the Southern portion of Ohio the road passes through Athens and Chillicothe, and runs into the Union depot at Cincinnati ; there the train will be transferred to the Queen and Crescent route, and passing through the famous blue grass region of Kentucky, will arrive early in the morning at Lexington, Ky.

Monday will be spent visiting the stock farms in the neighborhood of Lexington and inspecting some of the most famous stallions and brood mares in the country. The train will leave that evening, arriving in Chicago Tuesday morning, September 16th.

Tuesday, Wednesday and Thursday will be occupied with the meeting of the Association, an inspection of the stock yards and great slaughter-houses of Chicago, and by a visit to the stock farm, famous for its Percherons and draught horses, of Dunham & Co., in DuWayne County, Ill., and on Thursday night the train will leave Chicago arriving in Philadelphia and New York on Friday afternoon, September 19th.

The price of the round trip ticket, including Pullman Cars, will not exceed $42.50. A dining car will be attached to the train on which meals will be served at seventy-five cents each.

If a sufficient number of the members of the Association and their friends will avail themselves of this exceptionably pleasant week of travel, a special train will be run, which will make very much faster time, otherwise the Pullman cars containing the party will be attached to a regular train.

The Committee desires that every Eastern member of the Association, who can go to Chicago, will arrange to make it one week of his Summer's vacation, and will notify the chairman, Dr. W. H. Hoskins, 12 South 37th Street, Philadelphia, Pa., at an early date of his intention to do so.

As it will undoubtedly be a large and interesting meeting, and there will be a large number of Western veterinarians in attendance, who will become applicants for membership in the Association, a cordial invitation is extended to Eastern veterinarians, who are not members of the Association, to join the excursion above, and attend the meeting.

As several members of the Association have expressed a desire to take their wives and others of their families with them, one car will be assigned to those who are accompanied by ladies. On the return trip, the tickets will be good to stop off at Deer Park and Oakland, the charming Summer resorts in the mountains, overlooking five states, Maryland, Pennsylvania, Virginia, West Virginia and Ohio, which have been rendered popular recently by the residence of President Cleveland and President Harrison.

For those who can take an extra day or two, either for the hotel amusement of these delightful Southern Summer resorts, or who care for a little fishing and shooting in the mountains, at this season in their height, a delay at one of these places will make an agreeable termination of the week's holiday.

The title of the paper which will be presented by Dr. D. E. Salmon, Chief of the Bureau of Animal Industry, was incorrectly given in the last number of the Journal. The correct title is: "*Some Recent Researches in the Bacteriology of the Diseases of the Domesticated Animals.*"

Dr. Austin Peters, of Boston, chairman of the Committee on "Intelligence and Education," is expected to made a most thorough review of the subject placed in his hands, and it may be looked upon, practically, as one of the original papers which will be presented before the association. The subject of Dr. Liautard's paper has not yet been announced, but we understand that it will be upon a practical subject. With Dr. Huidekoper's paper on "Contracted Feet," and a probable paper from Dr. Tait Butler, ample scientific matter for discussion will be furnished.

We trust that the local and State veterinary associations will take up the subject of this meeting of the United States Veterinary Medical Association, and canvass their members in regard to their attendance at it, and notify the chairman of the committee at an early date of the probable number which will represent each society.

The resident State secretaries of the United States Veterinary Medical Association in the Eastern States are requested to constitute themselves, each a committee to arrange for the members of their respective States to join this party at New York or Philadelphia on the morning of September 14th.

The Baltimore and Ohio Railroad, over which this trip will be made, has been recently rebedded, which allows much comfort of travel at great speed, combined with safety. It passes through a rich and beautiful mountainous and agricultural section of the country, much of which is now being developed to be a great commercial interest.

The facilities will be seen, by which immense numbers of horses and cattle are handled, each month, from the Western side of the Ohio River to the Eastern States, which will be of direct interest to veterinarians both from a commercial and from a sanitary standpoint.

